# Cerebellar dysfunction in rodent models with dystonia, tremor, and ataxia

**DOI:** 10.3389/dyst.2023.11515

**Published:** 2023-12-08

**Authors:** Meike E. van der Heijden, Roy V. Sillitoe

**Affiliations:** 1 Department of Pathology & Immunology, Baylor College of Medicine, Houston, TX, United States,; 2 Jan and Dan Duncan Neurological Research Institute at Texas Children’s Hospital, Houston, TX, United States,; 3 Department of Neuroscience, Baylor College of Medicine, Houston, TX, United States,; 4 Program in Developmental Biology, Baylor College of Medicine, Houston, TX, United States,; 5 Development, Disease Models & Therapeutics Graduate Program, Baylor College of Medicine, Houston, TX, United States

**Keywords:** dystonia, tremor, cerebellum, rodent, ataxia

## Abstract

Dystonia is a movement disorder characterized by involuntary co- or over-contractions of the muscles, which results in abnormal postures and movements. These symptoms arise from the pathophysiology of a brain-wide dystonia network. There is mounting evidence suggesting that the cerebellum is a central node in this network. For example, manipulations that target the cerebellum cause dystonic symptoms in mice, and cerebellar neuromodulation reduces these symptoms. Although numerous findings provide insight into dystonia pathophysiology, they also raise further questions. Namely, how does cerebellar pathophysiology cause the diverse motor abnormalities in dystonia, tremor, and ataxia? Here, we describe recent work in rodents showing that distinct cerebellar circuit abnormalities could define different disorders and we discuss potential mechanisms that determine the behavioral presentation of cerebellar diseases.

## Introduction

Dystonia is a complex movement disorder that is characterized by involuntary muscle co- or over-contractions that cause abnormal and sometimes painful postures and movements [1]. Although dystonia is the third most common movement disorder in the United States [2], the neural mechanisms that give rise to these abnormal muscle contractions remain poorly understood [3–6]. This is, in part, due to the heterogeneity in the etiology, manifestation, and comorbidity of dystonia symptoms with other movement disorders including ataxia and tremor [7–13]. Further, few genetic mouse models of primary dystonia recapitulate the behavioral aspects of the human disease [14–16], although viral-, pharmacological-, and circuit-based mouse models do show overt dystonic movements and relevant abnormal postures [16–21].

Studies in patients and rodent models point to several brain regions as being associated with dystonia pathophysiology, with current work mainly implicating the somatosensory cortex, basal ganglia, and cerebellum [6, 16, 20–25]. These brain regions are functionally connected to each other, which makes it difficult to pinpoint a single origin of the symptoms and raises challenges for defining the ideal therapeutic targets with which to alleviate dystonic movements [23, 26–28].

Despite these hurdles, there is an emerging consensus that the cerebellum represents a central node in the circuit pathophysiology of dystonia and that cerebellar output neurons may even act as a “gate” in the manifestation of dystonic symptoms in animal models [3, 26, 29]. On the one hand, disrupting neural function in the cerebellum using genetics [18, 21], pharmacological compounds [19, 20, 30], and optogenetics [29] can cause dystonia in rodent models. On the other hand, neuromodulation [17, 21] and pharmacological silencing [20, 21] of the cerebellar circuit reduces dystonic symptoms in animal models. While these findings provide strong evidence that cerebellar pathophysiology drives dystonia, they also highlight a problem. Cerebellar pathophysiology is also strongly associated with movement disorders that have different motor disturbances, namely tremor [31–34] and ataxia [35–37]. Even though these three movement disorders can often occur together, they can also occur as independent conditions, which suggests that there may be overlapping features as well as unique pathophysiological signatures for dystonia, tremor, and ataxia [38, 39]. Yet, it remains unclear how dysfunction in one brain region, the cerebellum, can cause these three distinct motor disorders.

**FIGURE 1:**
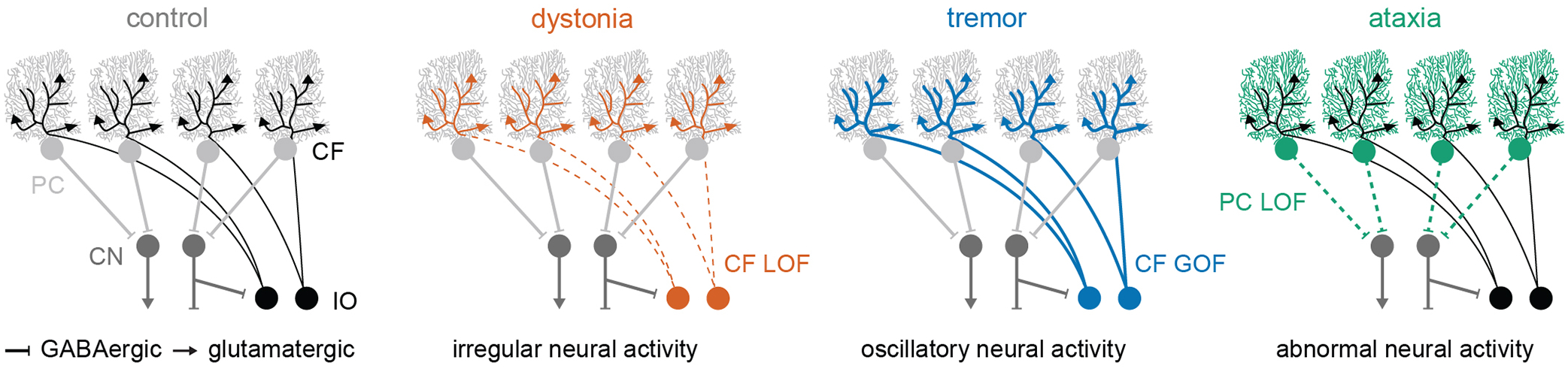
Schematic representation of the cerebellar circuit and the hypothesized neural abnormalities that are currently being tested in animal models of dystonia, tremor, and ataxia. Abbreviations: PC, Purkinje cell; CN, cerebellar nuclei; CF, climbing fiber; IO, inferior olive; LOF, loss-of-function; GOF, gain-of-function.

The cerebellum is comprised of a repeated circuit, which includes at least two sequential nodes of neural signal convergence [40, 41]. Excitatory and inhibitory afferent inputs converge onto the Purkinje cells in the cerebellar cortex, and then the Purkinje cells send convergent inhibitory projections to the predominant output neurons of the cerebellum, the cerebellar nuclei neurons (Figure 1). Thus, nearly all cerebellar signals are transmitted through the Purkinje cells and the cerebellar nuclei neurons. In the context of disease, dysfunction of both cell types has been observed in mouse models of cerebellar dystonia, tremor, and ataxia [21, 32, 42]. Therefore, the main cerebellar cell types, Purkinje cells and cerebellar nuclei neurons, are essential for normal motor function, and dysfunction of both cell types is implicated in all three motor disorders.

In this review, we discuss how cerebellar pathophysiology can potentially drive distinct motor phenotypes in rodent models. Namely, we propose that the presentation of cerebellar dysfunction is determined by how its neurons are affected. We focus our discussion on studies that have used *in vivo* manipulations of cerebellar function to devise animal models with dystonia, tremor, and ataxia.

## Cerebellar pathophysiology in dystonia

Dystonia is clinically diagnosed when involuntary muscle contractions that cause abnormal postures or limb positioning are present in affected individuals [1]. In rodents, dystonic symptoms manifest as abnormal postures that often include stiff, stretched out limbs, splayed paw digits, or a stiff tail, and these postures can be quantified with a modified dystonia rating scale [17]. Additional analyses can be performed with electromyography (EMG) to test the duration of muscle contractions or the presence of co- and over-contractions of opposing muscle pairs [17]. Here, we focus only on rodent models that have severe and overt dystonic symptoms that were quantified using the dystonia rating scale and/or EMG recordings conducted in behaving mice.

Some of the first indications of cerebellar dysfunction in dystonia came from studies in dystonic rats [43–46]. Cerebellar neurons in the dystonic rat, *dt*, showed abnormal irregular activity, and removing the cerebellum reduced the dystonic behaviors in these animals [47, 48]. Further studies using harmaline, a pharmacological compound that acts via the climbing fibers that synapse onto Purkinje cells, revealed that synaptic connectivity is impaired in the *dt* rat [45, 49]. Those studies provided three major discoveries that have since been replicated in other animal models, which we will further summarize below: 1) dystonia is associated with irregular spiking activity in the cerebellar circuit; 2) loss-of-function in cerebellar climbing fibers can drive dystonia; and 3) removing cerebellar function when circuit activity is irregular is behaviorally beneficial.

### Dystonia is associated with irregular spiking activity in the cerebellar circuit

Irregular spiking activity that presents as bursts in cerebellar firing is the most consistent finding across mouse models with overt cerebellar dystonia [19, 21, 30, 50–52]. Irregular spiking activity has been found in mouse models with local application of dystonia-inducing pharmacological compounds (kainic acid and ouabain) into the cerebellum [19, 30], downregulation of dystonia-associated genes using viral shRNA delivery within the cerebellum [50–52], and local circuit manipulations to cerebellar connectivity (elimination of neurotransmission at climbing fiber to Purkinje cell synapses) [21]. Despite the consistency in these findings, whether these irregular spiking patterns directly drive dystonic symptoms or whether the symptoms are caused by proprioceptive feedback from the muscles and limbs to the cerebellum remains unclear.

### Loss-of-function in cerebellar climbing fibers can drive dystonia

Loss of climbing fiber activity can drive dystonia. Similar to the earlier findings in the dystonic *dt* rat, where climbing fibers were functionally impaired, genetic elimination of neurotransmission from climbing fibers results in severe, early-onset dystonia [21, 45]. A more recent study has revealed that elimination of neurotransmission in only a subset of climbing fibers does not cause overt dystonic symptoms or abnormal muscle activity, suggesting the possible presence of a threshold for network dysfunction that must be surpassed to cause dystonic phenotypes [53]. However, this loss-of-function in cerebellar climbing fibers is not an obligatory requisite for initiating dystonia in animal models. For example, direct changes in Purkinje cell function that are induced through acute pharmacological application, genetic perturbations, or optogenetic stimulation also cause dystonic phenotypes in mice [18–20, 29, 30, 51, 52].

### Removing cerebellar function when circuit activity is irregular is behaviorally beneficial

A robust and consistent finding is that neuromodulation of cerebellar circuit activity can reduce dystonic symptoms. Specifically, silencing cerebellar nuclei neurons using muscimol normalizes dystonic symptoms in the ouabain model of dystonia and in mice lacking neurotransmission at climbing fiber synapses [20, 21]. In the *tottering* mouse model, which exhibits intermittent dystonic episodes, no such episodes are observed when Purkinje cell degeneration is induced using a secondary mutation [54]. Additionally, one study has shown that deep brain stimulation targeted to the cerebellar nuclei can significantly reduce dystonic symptoms in the genetic mouse model that lacks climbing fiber neurotransmission [21]. Thus, interfering with or reducing aberrant activity at the level of the cerebellar nuclei can improve behavior in dystonic mice.

## Cerebellar pathophysiology in tremor

Tremor is clinically defined as involuntary oscillatory or rhythmic movements in any body part [55, 56]. In animals, this phenotype can be measured using EMG recordings or accelerometer measurements of movements [32, 57–59]. The power, or severity, of the oscillations can be extracted using fast Fourier transform analyses. Although all animals and people exhibit some level of physiological tremors during movements, only those whose tremors significantly exceed baseline averages or have a change in frequency are described as having pathological tremor.

One of the classic models used to study tremor in animals is the pharmacologically-induced harmaline model [34, 60]. In rodents, harmaline rapidly initiates a severe tremor at approximately 10 Hz via the synchronized hyperactivity of inferior olive neurons, which communicate with the cerebellum through climbing fiber projections. Functional data generated from the harmaline models of tremor have provided the following three major discoveries: 1) tremor is associated with irregular, often oscillatory, activity within the cerebellum; 2) gain-of-function in climbing fiber activity can drive tremor; and 3) cerebellar nuclei neuromodulation can eliminate tremor.

### Tremor is associated with irregular, often oscillatory activity within the cerebellum

Purkinje cells and their downstream target nuclei neurons transition to burst activity after harmaline injection [32]. Burst activity was also found in the *Car8*^*wdl/wdl*^ mutant, where tremor, in addition to dystonia and ataxia, is caused by a loss-of-function mutation in the carbonic anhydrase 8 gene that is heavily expressed in Purkinje cells [33, 61–63]. Cerebellar neurons in *Car8*^*wdl/wdl*^ mice treated with propranolol, a drug frequently used to treat tremor patients, still display burst activity, but the firing rate within the burst is decreased [33]. The burst activity upon harmaline treatment is associated with oscillatory firing in the cerebellar cortex and nuclei, as measured by LFP recordings [64]. Similar oscillatory activity is observed in the *hotfoot17J* tremor mouse model [31]. Finally, inducing an irregular, oscillatory activity pattern in cerebellar neurons using optogenetics also causes tremor [31, 32]. Thus, irregular, oscillatory activity is a shared pathophysiology across tremor mouse models, and this activity is sufficient to drive tremor.

### Gain-of-function in climbing fiber activity can drive tremor

In rodents treated with harmaline, the oscillatory activity within the cerebellar circuit is induced by increased activity and synchrony of climbing fibers and their associated Purkinje cells [32, 34, 65, 66]. This climbing fiber activity is necessary for tremor, as inferior olive neuron silencing or climbing fiber loss eliminates the ability of harmaline to induce tremor [58, 65]. In the *hotfoot17J* mouse model, climbing fibers over-innervate Purkinje cells, which may contribute to tremor genesis, and reducing climbing fiber activity reduces tremor intensity [31]. Abnormal climbing fiber pruning during development can also lead to severe tremor in early postnatal mice [67]. Therefore, abnormal inferior olive activity, and thereby the altered function of climbing fiber activity, is strongly associated with tremor pathophysiology in animal models of the disease.

## Cerebellar nuclei neuromodulation can eliminate tremor

Multiple studies suggest that tremor can be caused by aberrant activity from the climbing fibers to Purkinje cells and via cerebellar nuclei neurons to other regions in the brain-wide motor network. If this hypothesis is correct, then tremor should be reduced or eliminated with neuromodulation of aberrant activity at each of these nodes. As discussed previously, climbing fiber-to-Purkinje cell activity is necessary for the propagation of harmaline-induced tremor [58, 65]. Additionally, impairing Purkinje cell neurotransmission prohibits the ability of harmaline to cause tremor [32]. Importantly, deep brain stimulation directed to the cerebellar nuclei neurons eliminates tremor in the harmaline mouse model [32] and in the *shaker* rat model [68]. These findings show that neuromodulation of the cerebellum effectively blocks tremor pathophysiology in rodents.

## Cerebellar pathophysiology in ataxia

Ataxia is clinically characterized by poor balance and the lack of coordination during movement. In the lab, ataxic phenotypes in models are often measured using motor assays like the accelerating rotarod or gait measurements such as foot printing or digit-gait [33, 69, 70]. Unlike EMG measurements or tremor measurements, motor assays like the accelerating rotarod are not ataxia-specific and can also be affected by other motor impairments [71, 72]. Changes in foot printing measurements can vary between ataxia models, but ataxic mice often have shorter steps with either a narrower or wider stance [33, 42, 73, 74]. Therefore, a combination of home-cage observations that examine mobility and behavior over extended periods of natural behavior, supplemented with a variety of motor assays, are best used for quantifying ataxic movements in animal models.

Ataxic mouse models were among the first genetic mouse models of disease studied, as their dramatic defects in gait readily distinguished them from control littermates [75, 76]. Prior to the availability of genetic sequencing, these spontaneous mutants were named according to their abnormal motor behaviors (e.g., *stumbler, lurcher, weaver, staggerer, tottering, waddles*) [61, 77–81]. Further research revealed that all of these spontaneous mutants have cerebellar alterations that lead to abnormal synaptic Purkinje cell innervation, Purkinje cell degeneration, or Purkinje cell dysfunction. After genetic sequencing and genetically engineered animals became available, mouse models with mutations that mimicked those in human patients with spinocerebellar ataxia exhibited similar Purkinje cell abnormalities [74, 82]. From these studies arose the following major complementary discoveries: 1) Purkinje cell loss-of-function is a common hallmark in ataxia; 2) ataxia is associated with abnormal Purkinje cell function; and 3) cerebellar nuclei neuromodulation reduces some of the behavioral symptoms that define ataxia.

### Purkinje cell loss-of-function is a common hallmark of ataxia

Purkinje cell degeneration is a common pathology found in mouse models of ataxia, which often arise from spontaneous genetic mutations [36, 37]. In fact, the loss of Purkinje cell function is thought to be a main driver of ataxia. Conditional elimination of Purkinje cell neurotransmission has confirmed that Purkinje cell loss-of-function, in the absence of neuronal loss, is sufficient to induce severe, early-onset ataxia [42]. In accordance with this model, not all mouse models with ataxia exhibit Purkinje cell loss; for example, no Purkinje cell loss is observed in the *waddles* mice (*Car8*^*wdl/wdl*^), with potentially restricted and patterned cell loss in older *tottering* and spinocerebellar ataxia type 6 mice [54, 62, 83–85].

### Ataxia is associated with abnormal Purkinje cell function

Regardless of whether an ataxia mouse model has or does not have Purkinje cell degeneration, it is the presence of Purkinje cell dysfunction that is strongly associated with abnormal motor phenotypes. In mouse models of spinocerebellar ataxia, Purkinje cells exhibit abnormal electrophysiological properties prior to, or closely aligned with, motor symptom onset [86–89]. In *tottering* and *waddles* mice, Purkinje cells exhibit irregular burst activity [33, 90]. Interestingly, *tottering* mice also exhibit intermittent dystonic attacks, and during these attacks, Purkinje cells exhibit faster and more irregular spiking activity than during the periods in which the mice predominantly display a milder ataxia [90, 91]. Similarly, the *waddles* mice exhibit tremor and dystonia in addition to ataxia, and when these mice are treated with a tremor reducing drug, Purkinje cell spike activity becomes slower and less irregular [33]. These observations suggest that the loss of normal Purkinje cell function can cause ataxia and that different types of Purkinje cell dysfunction (measure at level of spikes) may present as diverse motor comorbidities [29, 92].

### Cerebellar nuclei neuromodulation can reduce some symptoms that are associated with ataxia

Similar to the findings in models of dystonia and tremor, ataxia in animal models is associated with abnormal cerebellar spike activity. However, both a complete loss of Purkinje cell function and aberrant Purkinje cell function can cause ataxia in rodent models. As a result, specifically from the perspective of “normalizing” neuronal activity and behavior in mice, treating ataxic symptoms using drugs or neuromodulation has been more challenging than treating dystonic or tremor symptoms. Nevertheless, some studies show that partial normalization of Purkinje cell dysfunction using pharmacologic compounds can improve motor coordination in animal models with ataxia [63, 87, 93, 94]. Similarly, neuromodulation using deep brain stimulation has been beneficial in rodent models for ataxia [68], specifically when combined with exercise [69]. These findings underscore the broad benefits of cerebellar brain stimulation for restoring behavior in animal models of cerebellar movement disorders.

## Discussion

In this review, we discuss how cerebellar function is affected in rodent models with dystonia, tremor, and ataxia. We highlight evidence showing how cerebellar Purkinje cells and nuclei neurons are dysfunctional in animal models with motor symptoms that present predominantly as dystonia, tremor, or ataxia. Abnormal irregular activity in cerebellar neurons is a hallmark of all three disorders, although evidence points towards unique properties in the spike train activity of each disorder. For example, although burst activity is typically observed in dystonia, the spike train activity in mouse models with tremor is also bursty but it is also often highly rhythmic and oscillatory [31, 32]. The difference in spike train irregularity between dystonia and ataxia may fall within a range of relative defects separated by the degree of irregularity, with a higher average irregularity in the spike activity of cerebellar neurons in dystonia compared to ataxia [3]. A recent study by our group used optogenetics to confirm that these differences in activity can directly drive different behavioral presentations of cerebellar dysfunction [29].

Another important point of consideration is whether the differences in neural dysfunction could be used to distinguish dystonic tremor from dystonia or tremor alone. Our recent work showed that inducing a dystonia-associated spiking signature in cerebellar neurons resulted in dystonic symptoms, with a milder tremor than when we changed the spiking pattern to the tremor-signature [29]. These findings suggest that similar to the relationship observed between the spiking activity in ataxia and dystonia, the neuronal spiking activity in dystonia and tremor may also fall on a continuum of cellular behaviors. Further, the relative severity of neural dysfunction, as measured by specific domains (spike irregularity or spike rate), may be used to define the behavioral expression of cerebellar disease. Future studies could leverage a combination of mouse models and optogenetics to better define how the comorbidities in ataxia, dystonia, and tremor originate from the neural spike train activity in the cerebellum.

In addition to providing a better understanding of disease pathophysiology, studies that more deeply resolve the behavioral impact of neuronal spike alterations will be valuable for developing effective therapies to treat cerebellum-associated disorders, including dystonia. Although neuromodulation of the cerebellar nuclei is effective in reducing dystonic, tremorgenic, and ataxic symptoms in mouse models [21, 32, 69], it does not completely rescue all motor impairments that are present in each condition [21, 32, 69]. If the differences in cerebellar dysfunction between each disorder can be fully appreciated, neuromodulation paradigms can be optimized to normalize neural function without inadvertently inducing additional impairments. Combining these approaches with cell type-specific or closed-loop neuromodulation paradigms will further enhance the specificity of treatments and reduce unwanted side effects. Moreover, a more complete understanding of how the cerebellum interacts with other key nodes in the motor circuit in each condition will reveal additional points in the network that may be especially susceptible to the different diseases, which would provide a richer set of brain targets for neuromodulation and drug therapies.

Importantly, the presence of disease-specific neural signatures that are found in animal models must be validated in human patients to confirm the translatability of preclinical findings. Disease-specific signatures would provide biomarkers that could aid in the diagnosis of complex diseases and the personalized fine-tuning of neuromodulation parameters. With this approach, clinicians could use patient-specific treatments to target the symptoms of dystonia safely and effectively.
